# Early detection of myocardial infarction following blunt chest trauma by computed tomography: a case report

**DOI:** 10.1186/s12872-017-0496-3

**Published:** 2017-02-10

**Authors:** Thung-Lip Lee, Chin-Feng Hsuan, Chen-Hsiang Shih, Huai-Wen Liang, Hsing-Shan Tsai, Wei-Kung Tseng, Kwan-Lih Hsu

**Affiliations:** 1 Division of Cardiology, Department of Internal Medicine, E-Da hospital / I-Shou University, Kaohsiung, Taiwan; 2 Department of Internal Medicine, E-Da cancer hospital / I-Shou University, Kaohsiung, Taiwan

**Keywords:** Blunt chest trauma, Coronary dissection, Computed tomography, Myocardial infarction

## Abstract

**Background:**

Blunt cardiac trauma encompasses a wide range of clinical entities, including myocardial contusion, cardiac rupture, valve avulsion, pericardial injuries, arrhythmia, and even myocardial infarction. Acute myocardial infarction due to coronary artery dissection after blunt chest trauma is rare and may be life threatening. Differential diagnosis of acute myocardial infarction from cardiac contusion at this setting is not easy.

**Case presentation:**

Here we demonstrated a case of blunt chest trauma, with computed tomography detected myocardium enhancement defect early at emergency department. Under the impression of acute myocardial infarction, emergent coronary angiography revealed left anterior descending artery occlusion. Revascularization was performed and coronary artery dissection was found after thrombus aspiration. Finally, the patient survived after coronary stenting.

**Conclusion:**

Perfusion defects of myocardium enhancement on CT after blunt chest trauma can be very helpful to suggest myocardial infarction and facilitate the decision making of emergent procedure. This valuable sign should not be missed during the initial interpretation.

## Background

Blunt cardiac trauma encompasses a wide range of clinical entities, including myocardial contusion, cardiac rupture, valve avulsion, pericardial injuries, arrhythmia, and coronary artery injury leading to myocardial ischemia or infarction. Autopsy studies of blunt chest trauma (BCT) have revealed that injury to the heart is involved in 20%, and the coronary arteries in less than 2% [1].

According to a review of 77 published cases of myocardial infarctions caused by BCT, the most frequently injured vessel is left anterior descending artery (LAD) (71%), followed by right coronary artery (19%), left main artery (6%), and left circumflex artery (3%). Coronary artery occlusion and dissection were documented in 57% and 16% of cases respectively. Other traumatic coronary injuries including vascular rupture, focal spasm, fissuring of atherosclerotic plaque, external hematoma compression, coronary embolism and thrombosis due to intimal tears, can also result in myocardial infarction [2].

Here we demonstrate a case of acute myocardial infarction (AMI) due to coronary artery dissection after blunt chest trauma, with computed tomography (CT) detected myocardium enhancement defect early at emergency department and the patient survived after coronary stenting.

## Case presentation

**Fig. 1:**
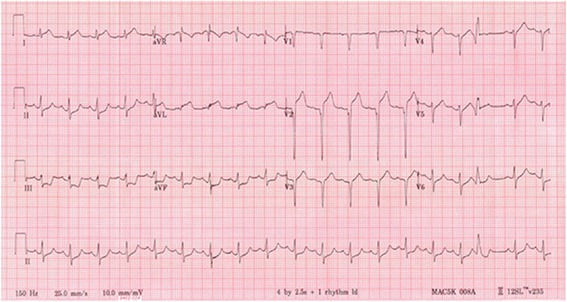
ECG showed QS pattern with ST segment elevation at lead V2 to V3 and reciprocal ST segment depression at leads II, III and aVF

**Fig. 2:**
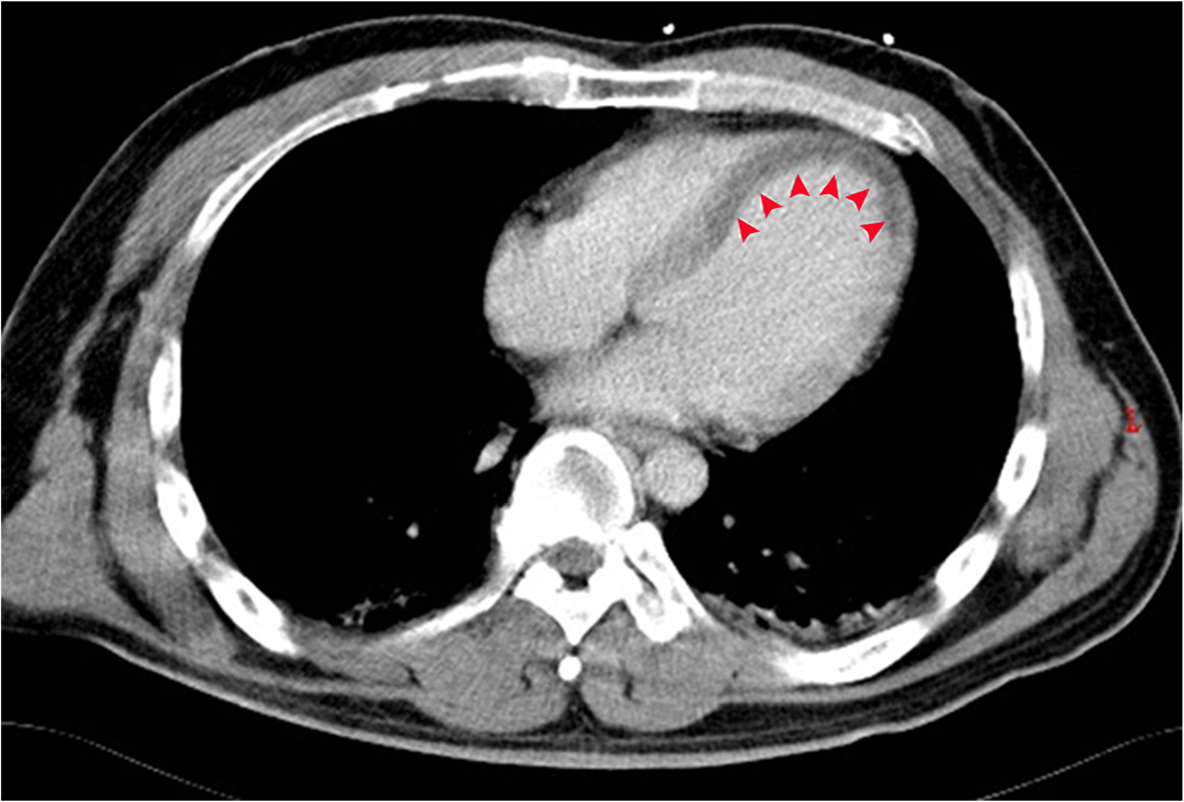
Contrast-enhanced CT revealed decreased myocardial enhancement over the interventricular septum and the left ventricular apex (*arrowhead*)

**Fig. 3:**
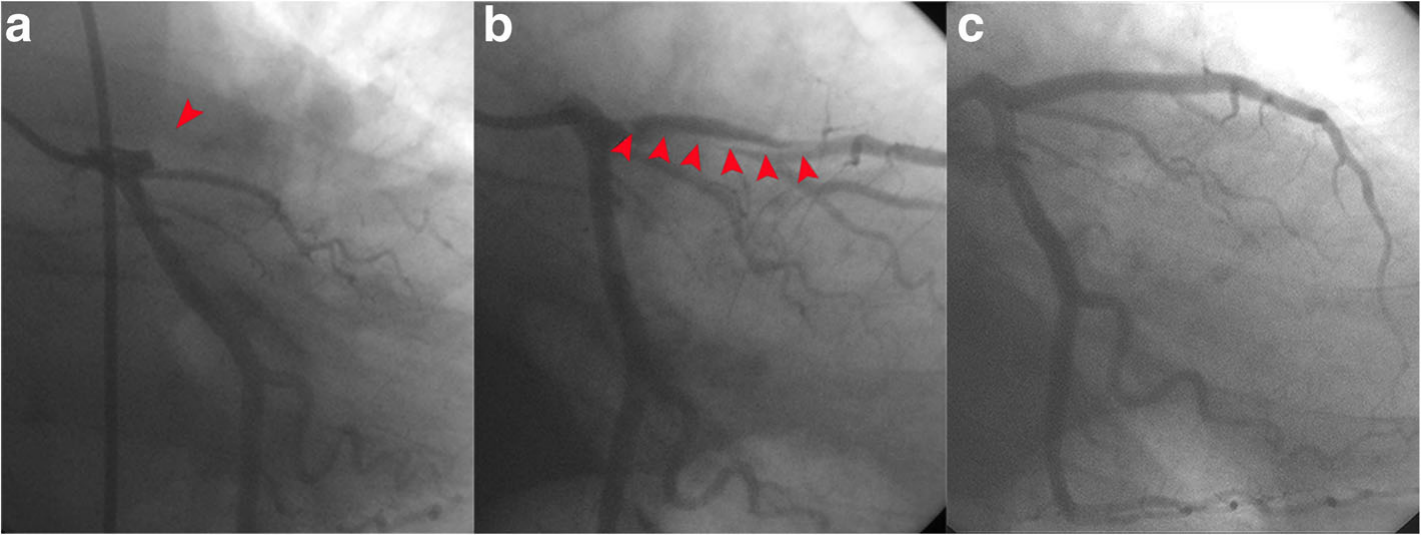
**a** Coronary angiography revealed total occlusion of the proximal LAD with a sharp end (*arrowhead*). **b** A long dissection flap (*arrowhead*) appeared at the occlusion site after thrombus aspiration. **c** Final angiography showed optimal stenting result of LAD

A 41-year-old man without any cardiovascular risk factors had a collision with a tree trunk while riding a motorcycle. Consciousness disturbance was noted at the emergency department one hour after the accident, physical examination revealed facial laceration and multiple sites trauma including ecchymosis over the chest wall. Whole body contrast-enhanced CT showed bilateral frontal subdural hematoma, multiple craniofacial bone fractures, left anterior rib fracture, and liver laceration with hemoperitoneum. Hypotension developed two hours after arrival with poor response to initial fluid resuscitation. Electrocardiogram (ECG) showed QS pattern with ST segment elevation at lead V2 to V3, ST segment elevation at lead I and aVL, and reciprocal ST segment depression at leads II, III and aVF (Fig. 1). CT was re-evaluated and a decreased myocardial enhancement over the interventricular septum and apex of the left ventricle was noted (arrowhead, Fig. 2). Under the impression of anterior AMI, coronary angiography showed total occlusion of the LAD (arrowhead, Fig. 3a). A dissection flap appeared at the occlusion site after thrombus aspiration (arrowhead, Fig. 3b). Two Driver bare metal stents of 4.0*30 and 3.5*24 mm were implanted to ensure full coverage of the dissection. The final angiography revealed optimal result with TIMI III flow (Fig. 3c). Patient’s hemodynamics was stable after the procedure and his consciousness improved gradually. No anticoagulant was prescribed due to high bleeding risk, and the subsequent hospital stay was uneventful. Echocardiography performed after the procedure revealed akinesis over the anterior wall of the left ventricle. The highest levels of cardiac enzymes were CK 4839 U/L, CK-MB 468.7 U/L, and hsTroponin-I 178.2 ng/ml. The patient discharged 14 days later with daily regimen of clopidogrel 75 mg and acetylsalicylic acid 100 mg for 3 months, then acetylsalicylic acid alone for another 6 months. No angiotensin-converting-enzyme inhibitor or beta-blocker was prescribed due to his relative low blood pressure, and neither statin prescribed due to his normal lipid level and non-atherosclerotic cause of myocardial infarction. In a 12-month follow-up the patient is asymptomatic, without any heart failure or recurrent symptom of myocardial ischemia.

## Discussion

The diagnosis of cardiac contusion in patients with BCT remains controversial, and early recognition of AMI from cardiac contusion is not easy. In cases of suspicious AMI, incremental myocardial damage may occur if the essential revascularization is delayed. Emergent coronary angiography with potential angioplasty is the preferred method of evaluation and management.

Symptoms such as chest pain in patients with AMI may mimic a concomitant rib fracture or muscular injury. Although a meta-anaysis supports the use of ECG and cardio-biomarker for the diagnosis of clinically significant myocardial injuries [3], ST segment abnormalities after BCT can be caused by cardiac contusion and pericarditis as well [4]. Troponin is the golden standard for diagnosis of myocardial infarction according to the new definition [5]. However, Mahmood I, et al. reported that one-fifth of patients with BCT had elevated troponin level even in the absence of clinical evidence of acute cardiac involvement [6]. Echocardiography may show regional abnormalities in wall motion in patients with myocardial contusion, but it cannot reliably distinguish cardiac contusion from ischemia associated with coronary artery dissection, either [4]. None of the above single examination alone is reliable for detection of myocardial infarction after BCT. Integrating information from multiple tests including ECG, echocardiography, cardiac biomarkers, and other image modalities like CT, is the key to early recognition of the event.

Magnetic resonance imaging (MRI) was reported to detect coronary artery anomalies and AMI after BCT [7, 8]. Delayed enhanced cardiac MRI can also detect the coronary wall hematoma, pericardial tears and the extent of post-traumatic AMI, as well as differentiate between contusion and AMI [9]. However, MRI plays lesser role during acute BCT setting due to long scanning time and challenges related to patients with polytrauma and unstable hemodynamics [10].

Currently, multidetector computerized tomography (MDCT) is the ideal imaging tool for the diagnosis and delineation of coronary artery anomalies in the population with relative low cardiovascular risk and low pretest probability of CAD population. MDCT may indicate for evaluate patient with BCT, and enables a comprehensive assessment of thoracic lesions including chest wall trauma, injured aorta, pericardial injury, coronary injury and cardiac rupture [10]. There are also some literature reported AMI diagnosed by MDCT after BCT with relative stable condition [8, 11]. However, coronary angiography instead of MSCT is preferred for higher risk group of population and in the setting of acute coronary syndrome, except for some specific indications, for examples coronary anomalies like anomalous origin and giant aneurysm [12].

CT is well described as a routine imaging modality for detecting thoracic injuries caused by BCT [13, 14]. Improvement in imaging technology had permitted the demonstration of the focal decreased ventricular myocardial enhancement in a specific coronary artery distribution, consistent with myocardial infarction [15]. There is report demonstrated focal myocardial enhancement defect on CT in a case of delayed diagnosed myocardial infarction after BCT [16]. In this present case, we showed early detection of myocardial enhancement defect on CT and subsequent immediate coronary revascularization with good clinical outcome.

## Conclusion

AMI due to coronary artery dissection after BCT is rare and may be life threatening. Perfusion defects of myocardium enhancement on CT can be very helpful to suggest myocardial infarction and facilitate the decision making of emergent procedure. This valuable sign should not be missed during the initial interpretation.

### Limitation

Coronary optical coherence tomography and intravascular ultrasound can provide further informative assessment of the dissected coronary artery, and facilitated the selection of size and length of the stent. In this case, we did not perform these procedures due to the unstable hemodynamic condition and no insurance coverage of the procedures.

## Abbreviations

AMI: Acute myocardial infarction
BCT: Blunt chest trauma
CT: Computed tomography
ECG: Electrocardiogram
LAD: Left anterior descending artery
MDCT: Multidetector computerized tomography
MRI: Magnetic resonance imaging
